# The Associations Between Cultural Identity and Mental Health Outcomes for Indigenous Māori Youth in New Zealand

**DOI:** 10.3389/fpubh.2018.00319

**Published:** 2018-11-13

**Authors:** Ashlea D. Williams, Terryann C. Clark, Sonia Lewycka

**Affiliations:** The School of Nursing, Faculty of Medical Health Sciences, University of Auckland, Auckland, New Zealand

**Keywords:** Māori, youth, cultural identity, mental health, ethic discrimination, indigenous, suicide, cultural programming

## Abstract

**Objectives:** To explore the relationships between Māori cultural identity, ethnic discrimination and mental health outcomes for Māori youth in New Zealand.

**Study Design:** Nationally representative, anonymous cross-sectional study of New Zealand secondary school students in 2012.

**Methods:** Secondary analysis of Māori students (*n* = 1699) from the national Youth'12 secondary school students survey was undertaken. Theoretical development and exploratory factor analysis were undertaken to develop a 14-item Māori Cultural Identity Scale (MCIS). Māori students reporting > 8 items were classified as having a strong MCIS. Prevalence of indicators were reported and logistic regression models were used to explore how wellbeing (WHO-5), depressive symptoms (Reynolds Adolescent Depression Scale-SF), and suicide attempts were associated with the MCIS.

**Results:** After adjusting for age, sex, ethnic discrimination and NZ Deprivation Index (NZDep), a strong Māori cultural identity (MCIS) was associated with improved wellbeing scores (OR 1.53, 95% CI 1.18–2.01) and fewer depressive symptoms (OR 0.53, 95% CI 0.38–0.73). Experiencing discrimination was associated with poorer wellbeing scores (OR 0.50, 95% CI 0.39–0.65), greater depressive symptoms (OR 2.2, 95% CI 1.55–3.18), and a previous suicide attempt (OR 2.47, 95% CI 1.71–3.58). Females less frequently reported good (WHO-5) wellbeing (OR 0.33, 95% CI 0.26–0.42), increased (RADS-SF) depressive symptoms (2.61, 95% CI 1.86–3.64) and increased suicide attempts [OR 3.35 (2.07–5.41)] compared to males. Wellbeing, depressive symptoms and suicide attempts did not differ by age or neighborhood level socio-economic deprivation, except those living in neighborhoods characterized as having medium level incomes, were less likely to have made a suicide attempt (OR 0.49, 95% CI 0.27–0.91).

**Conclusions:** Māori youth who have a strong cultural identity were more likely to experience good mental health outcomes. Discrimination has a serious negative impact on Māori youth mental health. Our findings suggest that programmes, policies and practice that promote strong cultural identities and eliminate ethnic discrimination are required to improve mental health equity for Māori youth.

## Introduction

Rangatahi Māori, the indigenous youth of New Zealand experience significantly poorer mental health outcomes compared to Pākehā/NZ European youth (1–7). Rates of serious depressive symptoms are similar (Māori 13.9%, NZ European 12.1%), but Māori youth report considerably higher prevalence of suicide attempts (Māori 6.5%, NZ European 2.7%) and poorer general wellbeing (Māori 10.5%, NZ European 6.8%) compared to their peers (1). Suicide death rates for Māori youth are almost twice that of non-Māori (Māori suicide 17.8 people per 100,000, non-Māori 10.6 per 100,000) (8). Despite the concerning health and social impacts of poor mental health, Māori youth are significantly less likely to be able to access healthcare when needed (1) and frequently experience mental health stigma (9) and ethnic discrimination (10).

Given the well documented mental health disparities of Māori youth, there remains little published evidence on why these disparities persist or how best to address them. Measuring the impact of structural racism, inter-generational trauma and the colonizing stress on youth remains complex. Lawson-Te Aho (11) suggests the impact of colonization for contemporary Māori youth has contributed to a breakdown of traditional cultural structures; leaving a legacy of hopelessness and loss of meaning. She also asserts that when “kinship-based cultural identity is intact and relationships are positive and functional, suicide can be prevented” (11, p.128). While there is evidence that whānau and community relationships are protective for mental health concerns (12–15) there is much less evidence to support the assertion that Māori cultural identity is also protective (16, 17).

There is conflicting literature regarding the positive influence of cultural identity on health outcomes for Māori. Brougham and Harr (18) found that high levels of collectivism, cultural knowledge and language led to improved mental wellbeing. Coupe's (19) study also found that a secure Māori identity, healthy connections to social groups and a sense of belonging were protective against suicide. However, Broughton as cited in Tatz (20) reported that some Māori youth who were immersed in Māori culture, still died from suicide. In contrast, Marie et al. (21) found that sole Māori ethnicity was associated with increased exposure to childhood physical abuse and inter-parental violence. Given the lack of cohesive literature and evidence in this area, the aim of this project was to identify whether a strong Māori cultural identity was associated with improved wellbeing for Māori youth.

## Materials and methods

Youth'12 is a nationally representative, anonymous cross-sectional survey of New Zealand secondary school students aged approximately 12–19 years old. The survey utilized a two-stage cluster sample design, with a third of all secondary schools in New Zealand invited to participate and then 20% of each participating school's roll was randomized and invited to participate. This accounted for approximately 3–4% of the total NZ secondary school population. The school response rate was 72.8% and student response rate was 68% (22). Ethical approval was gained from the University of Auckland Human Participants Ethics Committee (UAHPEC) (Ref 2011/206).

The Youth'12 questionnaire administered via Multi-Media Computer Administered Survey Instrument (MCASI) (23) on internet tablets was based on the Youth2000 survey series questionnaires in 2001 and 2007 available at www.youthresearch.auckland.ac.nz. There were nine main domains in the survey that addressed the wider determinants of wellbeing for NZ youth including variables relevant to this paper; ethnic specific questions, mental health, family wellbeing and social connections. The questionnaire was administered in English and te reo Maori (Māori language) options.

### Demographic measures

These were based on self-report for age, sex (“male” and “female” only) and ethnicity. Ethnicity was based on the New Zealand census standard 2001/2006 ethnicity question: “Which ethnic group do you belong to?” Students were able to choose more than one response from the list of 23 options used in the statistical standard for ethnicity (24). We have utilized the New Zealand ethnic prioritization method (25). Prioritized ethnicity is based on the hierarchy; Māori > Pacific > Asian > Other > New Zealand European (NZE). NZDep combines variables from census data related at the neighborhood level to income, home ownership, financial support, employment, qualifications, living space, communication and transport, to reflect levels of deprivation (26).

### MCIS scale development

A literature review was undertaken to identify the components of Māori cultural identity. Relevant research came from a selection of journal articles, gray literature, books and Māori Health Models. Four themes were identified: (1) *Whanaungatanga (Collectivist Identity and Relationships)* includes active participation and sense of belonging to family and various social groups (27–34). (2) *Ko wai au? (Who am I?/Personal Ethnic Identity)* is the concept of who you are and where you come from spiritually, mentally and physically (27–37). (3) *Ngā Taonga Māori (Precious Māori Cultural Resources)* includes the accessibility and participation in traditional knowledge and practices (28–34, 38–40). Lastly are the (4) *Social Determinants of Wellbeing* that might include socio-economic factors, history, colonization, structural, interpersonal or internalized discrimination, policy, changing demographics and media representations (12, 28, 29, 31, 33, 35, 39, 41–43). For the purposes of these analyses, the two themes Ko wai au? and Ngā Taonga Māori were utilized to guide our development of the Māori Cultural Identity Scale (MCIS) utilizing available questions from the Youth'12 national health and wellbeing survey questionnaire (**Table 2**).

Distributions of variables were explored, and we excluded those with high levels of missing data. Correlations between all of the variables were explored through correlation matrices, and we excluded further variables with low correlations to any of the others. With the remaining 14 cultural variables, we explored how these clustered together using factor analysis. We created a MCIS with a range of 0–14 by summing the number of variables present for each student. We divided students into two groups using the median, and those who reported higher than the median, were coded as “high cultural identity” (8 or more items present) and “low cultural identity” (7 or fewer items).

We included the theme Social Determinants of Wellbeing as covariates and possible confounders in our analysis through the inclusion of deprivation and discrimination. *Neighborhood deprivation* was measured using The New Zealand Deprivation Index (NZDep2006). (26), and a *discrimination* variable was constructed from three questions based on earlier work (10): “Have you ever been treated unfairly (e.g., treated differently) by a teacher because of your ethnicity or ethnic group? Have you ever been treated unfairly (e.g., treated differently, kept waiting) by a health professional (e.g., doctor, nurse, dentist etc.) because of your ethnicity or ethnic group? Have you been treated unfairly (picked on, hassled, etc.) by the police because of your ethnic group?” Responses were binary (yes/no) if they had experienced any discrimination.

### Dependent variables

Three mental health outcomes utilized in these analyses were: The 5-item World Health Organization Well-Being Index (WHO-5), a valid and reliable measure of positive quality of life, with a cut-off of 13 or higher being a measure of good, very good or excellent wellbeing (44); The Reynolds Adolescent Depression Scale- Short Form (RADS- SF), a 10 item valid and reliable self-report survey, based on the DSM-III diagnostic criteria for major depressive disorder and dysthymic disorder, along with symptoms from the Research Diagnostic Criteria for Affective Disorders (45); and suicide attempt, based on one question “During the last 12 months have you tried to kill yourself (attempt suicide)?” with binary response (yes/no).

### Analyses

Logistic regression models were utilized, to explore the associations between MCIS and the three mental health outcomes. We adjusted these models sequentially for age and sex, neighborhood deprivation, and discrimination. We explored for effect modification by age and sex by including interaction terms in models. Results are presented as weighted percentages, odds ratios with 95% confidence intervals.

## Results

Of the 91 schools that participated, 87 had Māori students, and of the 8,500 participating students, 1,701 identified as Māori. Māori youth comprise approximately 19.7% of the total New Zealand secondary school population in 2011 and the Youth'12 survey achieved a representative sample of 20.1% Māori. Māori students who participated in the Youth'12 survey had a slightly higher proportion of females (52.8%), and a higher proportion of students were 15 years or younger (70.8%) (Table 1). Māori were more likely to be living in areas with higher levels of deprivation, with 80.2% of the sample living in medium or highly deprived areas. A quarter (25.5%) of all Māori participants reported that they had experienced some form of ethnic discrimination at school, in health care or with the police. There were missing data from 27 participants (1.6%) for variables included in the analyses (age, sex, NZ Dep, discrimination and cultural identity), and these were excluded from all analyses. In terms of mental health outcomes, 75.1% of students reported good wellbeing based on WHO-5 wellbeing scale (*n* = 1,219/1,623), 13.8% report significant depressive (RADS-SF) symptoms (*n* = 219/1,591) and 6.5% reported a suicide attempt in the previous twelve months (*n* = 1,071,638).

**Table 1:** Descriptive participant characteristics by level of cultural identity (unweighted).

	**Total sample (including māori) *n*(%)**	**Total māori *n*(%)**	**High cultural identity *n*(%)**	**Low cultural identity *n*(%)**
**AGE**				
≤13 years	1,838 (21.7)	406 (24.3)	219 (26.7)	187 (21.9)
14 years	1,896 (22.3)	429 (25.6)	226 (27.6)	203 (23.8)
15 years	1,755 (20.7)	350 (20.9)	168 (20.5)	182 (21.3)
16 years	1,578 (18.6)	274 (16.4)	124 (15.1)	150 (17.6)
≥17	1,422 (16.8)	215 (12.8)	83 (10.1)	132 (15.5)
**SEX**				
Male	3,874 (45.6)	790 (47.2)	373 (45.5)	417 (48.8)
Female	4,623 (54.4)	884 (52.8)	447 (54.5)	437 (51.2)
**NZ DEP**				
Low deprivation	2,718 (32.4)	331 (19.8)	107 (13.1)	224 (26.2)
Medium	3,001 (35.8)	571 (34.1)	244 (29.8)	327 (38.3)
High deprivation	2,674 (31.9)	772 (46.1)	469 (57.2)	303 (35.5)
**ANY ETHNIC DISCRIMINATION**				
No	NA	1,247 (74.5)	551 (67.2)	696 (81.5)
Yes		427 (25.5)	269 (32.8)	158 (18.5)
	8,500	1,674	820 (49.0)	854 (51.0)

In general, there were high levels of Māori cultural identity concepts evident in the Youth'12 survey; *Ngā Taonga Māori* (*Precious Māori Cultural Resources*) and *Ko wai au?* (*Who am I?/Personal Ethnic Identity*) (Table 2). Items in these two themes were highly correlated, and we combined them into one Māori Cultural Identity Scale (MCIS). Using the MCIS scale, 49.0% of participants reported having eight or more cultural identity variables (Table 1). Younger students, female students and those living in areas of high deprivation reported higher levels of cultural identity. More students who had high cultural identity had reported experiences of discrimination. There was no evidence for effect modification of the association between cultural identity and mental health outcomes by age or sex.

**Table 2:** Questions from the Youth'12 survey utilized to develop the Maori cultural identity scale.

	**Survey question**	**Response**	***n/N***	**%**	**95% CI**
Ngā Taonga Māori	How satisfied are you with your knowledge of things Māori?	Very satisfied and satisfied	770/1,691	45.3	42.2–48.4
(Precious Māori Cultural Resources)	Do you know your iwi (tribe or tribes)?	Yes	1,303/1,700	76.6	74.4–78.8
	Have you learnt about Māori culture, such as language, songs, cultural practice or family history/ancestry (whakapapa)?	Yes	1,514/1,700	89.1	87.3–90.8
	Have you ever been to a tangi or unveiling?	Yes	1,335/1,700	78.5	74.6–82.4
	What languages do your parents or the people who look after you usually speak?	Māori	566/1,701	32.9	28.9–37.0
	Which languages can you speak well enough to have a conversation in?	Māori	448/1,700	26.1	23.6–28.7
	How well are you personally able to speak Māori in day-to- day conversation? By this we mean more than a few words or phrases?	Very well, Well and Fairly well	787/1,699	46.0	43.2–48.9
	How well are you personally able to speak Māori in day-to- day conversation? By this we mean more than a few words or phrases?	Very well, Well and Fairly well	530/1,698	31.0	28.4–33.7
Ko wai au? (Personal Ethnic Identity)	Which is your main ethnic group (the one you identify with most)?	Māori	839/1,660	50.2	46.4–54.0
	Are Māori values important to you (e.g., Whanau and Hui; Family gatherings), Karakia (prayer), Wairua (Spirituality) or Whakapapa (Family history)?	Very important	662/1,697	38.8	34.9–42.6
	Are you proud of being Māori?	I'm very proud	1,214/1,694	71.3	68.3–74.4
	How often do you think about your ethnicity or ethnic group?	Often and all the time	671/1,701	39.3	36.3–42.2
	Earlier you told us about your ethnicity. Now we would like to know about other people's reaction to your ethnicity. How do other people usually classify your ethnic group (i.e., what ethnic group/s do other people usually think you are)? (you may choose as many as you need)	Māori	1,130/1,701	66.3	63.0–69.5
	How important is it to you to be recognized as a Māori person?	Very important and Important	984/1,694	57.9	54.3–61.4

In univariate analyses, there was a strong association between Māori Cultural Identity and WHO-5 Wellbeing scores (OR 1.37 95% CI 1.07–1.76) (Table 3, Model 1). Being younger, male and poorer was also associated with higher WHO wellbeing scores, while experience of discrimination was associated with lower wellbeing (OR 0.63, CI 0.50–0.79). Adjusting for age, sex, and NZ Deprivation, did not alter the relationship between cultural identity and wellbeing (Table 3, Models 2 and 3). However, after adjusting for experience of discrimination (Table 3, Model 4), the relationship between cultural identity and wellbeing became stronger, suggesting that discrimination confounded the association, with those reporting high cultural identity also being more likely to report discrimination, and those reporting discrimination being more likely to report poor wellbeing.

**Table 3:** Associations between Maori cultural identity and WHO wellbeing score.

	**Wellbeing *n* (%)**	**Model 1 OR (95% CI)**	**Model 2 aOR (95% CI)**	**Model 3 aOR (95% CI)**	**Model 4 aOR (95% CI)**
**M**$\bar{\text{A}}$**ORI CULTURAL IDENTITY**					
Low cultural identity	604/835 (72.3)	1	1	1	1
High cultural identity	615/788 (78.1)	1.37 (1.07–1.76)	1.41 (1.09–1.83)	1.37 (1.06–1.77)	1.53 (1.18–2.01)
**AGE**					
≤13 years	308/386 (79.8)	1	1	1	1
14 years	305/415 (73.5)	0.71 (0.50–1.00)	0.70 (0.49–0.98)	0.69 (0.49–0.98)	0.70 (0.49–1.00)
15 years	265/344 (77.0)	0.84 (0.61–1.17)	0.86 (0.62–1.19)	0.86 (0.62–1.19)	0.88 (0.63–1.22)
16 years	186/269 (69.1)	0.57 (0.38–0.85)	0.57 (0.38–0.84)	0.57 (0.38–0.85)	0.60 (0.40–0.89)
≥17	155/209 (74.2)	0.73 (0.50–1.05)	0.79 (0.53–1.18)	0.80 (0.54–1.18)	0.80 (0.54–1.21)
**SEX**					
Male	643/760 (84.6)	1	1	1	1
Female	576/863 (66.7)	0.36 (0.29–0.46)	0.35 (0.28–0.45)	0.36 (0.28–0.45)	0.33 (0.26–0.42)
**NEW ZEALAND DEPRIVATION INDEX**					
Low deprivation (1–3)	231/322 (71.7)	1		1	1
Medium deprivation (4–7)	414/558 (74.2)	1.12 (0.80–1.58)		1.12 (0.80–1.58)	1.12 (0.80–1.57)
High deprivation (8–10)	574/743 (77.3)	1.33 (1.00–1.77)		1.20 (0.88–1.63)	1.22 (0.91–1.65)
**ANY ETHNIC DISCRIMINATION**					
No	943/1,218 (77.4)	1			1
Yes	276/405 (68.2)	0.63 (0.50–0.79)			0.50 (0.39–0.65)
Total	1,219/1,623 (75.1)				

*Model 1, Unadjusted model for associations with WHO wellbeing; Model 2, Model adjusted for age and sex; Model 3, Model adjusted for age, sex and NZ Dep; Model 4, Model adjusted for age, sex, NZ Dep and discrimination*.

In univariate analyses looking at the association between Māori Cultural Identity and depressive symptoms, high cultural identity was associated with lower levels of depression (Table 4, Model 1). Female students (OR 2.31, CI 1.69–3.17) and those who experienced discrimination (OR 1.72, CI 1.24–2.40) were much more likely to have depressive symptoms. Similar to WHO-5 wellbeing, adjusting for age, sex and NZ Dep had little effect on the association between cultural identity and depressive symptoms (Table 4, Models 2 and 3), while adjusting for experience of discrimination strengthened the relationship (OR 0.53, CI 0.38-0.73) (Table 4, Model 4).

**Table 4:** Associations between Maori Cultural Identity and symptoms of depression (using RADS-SF score).

	**Depressive symptoms *n* (%)**	**Model 1 OR (95% CI)**	**Model 2 aOR (95% CI)**	**Model 3 aOR (95% CI)**	**Model 4 aOR (95% CI)**
**M**$\bar{\text{A}}$**ORI CULTURAL IDENTITY**					
Low cultural identity	135/824 (16.4)	1	1	1	1
High cultural identity	84/767 (11.0)	0.62 (0.47–0.82)	0.58 (0.43–0.77)	0.60 (0.44–0.81)	0.53 (0.38–0.73)
**AGE**					
≤13 years	49/383 (12.8)	1	1	1	1
14 years	62/408 (15.2)	1.23 (0.82–1.84)	1.24 (0.82–1.86)	1.25 (0.83–1.87)	1.24 (0.81–1.89)
15 years	56/331 (16.9)	1.40 (0.91–2.14)	1.36 (0.89–2.09)	1.37 (0.89–2.11)	1.36 (0.87–2.11)
16 years	33/266 (12.4)	0.94 (0.60–1.47)	0.90 (0.56–1.44)	0.90 (0.56–1.44)	0.84 (0.52–1.36)
≥17	19/203 (9.36)	0.69 (0.39–1.20)	0.60 (0.34–1.07)	0.60 (0.34–1.07)	0.58 (0.32–1.04)
**SEX**					
Male	64/734 (8.7)	1	1	1	1
Female	155/857 (18.1)	2.31 (1.69–3.17)	2.41 (1.76–3.31)	2.41 (1.75 (3.32))	2.61 (1.86–3.64)
**NEW ZEALAND DEPRIVATION INDEX**					
Low deprivation (1–3)	53/320 (16.6)	1		1	1
Medium deprivation (4–7)	78/547 (14.3)	0.84 (0.59–1.17)		0.84 (0.59–1.19)	0.84 (0.59–1.18)
High deprivation (8–10)	88/724 (12.2)	0.70 (0.47–1.03)		0.76 (0.51–1.14)	0.73 (0.49–1.09)
**ANY ETHNIC DISCRIMINATION**					
No	144/1,201 (12.0)	1			1
Yes	75/390 (19.2)	1.72 (1.24–2.40)			2.22 (1.55–3.18)
Total	219/1,591 (13.8)				

*Model 1, Unadjusted model for associations with WHO wellbeing; Model 2, Model adjusted for age and sex; Model 3, Model adjusted for age, sex and NZ Dep; Model 4, Model adjusted for age, sex, NZ Dep and discrimination*.

In univariate analyses looking at the association between Māori Cultural Identity and attempted suicide, there was no evidence for an association (Table 5, Model 1). Only female students (OR 2.96, CI 1.88–4.65) and those experiencing discrimination were more likely to have attempted suicide (OR 2.23, CI 1.59–3.14). Those students living in neither low nor high deprivation areas were less likely to have made a suicide attempt (OR 0.54, CI 0.29–1.00). After adjusting for age, sex and deprivation there remained no evidence for an association between cultural identity and suicide attempt (Table 5, Models 2 and 3). Adjusting for discrimination had a larger effect on the association between cultural identity and suicide attempts, but the relationship was still not significant (OR 1.14, CI 0.71–1.84) (Table 5, Model 4).

**Table 5:** Associations between Maori cultural identity and attempted suicide within the previous 12 months.

	**Attempted suicide *n* (%)**	**Model 1 OR (95% CI)**	**Model 2 aOR (95% CI)**	**Model 3 aOR (95% CI)**	**Model 4 aOR (95% CI)**
**M**$\bar{\text{A}}$**ORI CULTURAL IDENTITY**					
Low cultural identity	46/840 (5.5)	1	1	1	1
High cultural identity	61/798 (7.6)	1.42 (0.93–2.16)	1.34 (0.88–2.03)	1.34 (0.84–2.13)	1.14 (0.71–1.84)
**AGE**					
≤13 years	24/399 (6.0)	1	1	1	1
14 years	35/418 (8.4)	1.37 (0.78–2.40)	1.41 (0.82–2.45)	1.43 (0.82–2.47)	1.39 (0.80–2.43)
15 years	25/344 (7.3)	1.20 (0.67–2.14)	1.22 (0.68–2.19)	1.25 (0.70–2.22)	1.19 (0.65–2.16)
16 years	12/269 (4.5)	0.73 (0.33–1.62)	0.76 (0.34–1.67)	0.77 (0.35–1.70)	0.72 (0.32–1.60)
≥17	11/208 (5.3)	0.83 (0.41–1.71)	0.85 (0.41–1.74)	0.86 (0.42–1.77)	0.85 (0.41–1.75)
**SEX**					
Male	26/769 (3.4)	1	1	1	1
Female	81/869 (9.3)	2.96 (1.88–4.65)	2.96 (1.87–4.68)	3.02 (1.91–4.79)	3.35 (2.07–5.41)
**NEW ZEALAND DEPRIVATION INDEX**					
Low deprivation (1–3)	26/324 (8.0)	1		1	1
Medium deprivation (4–7)	25/563 (4.4)	0.54 (0.29–1.00)		0.49 (0.26–0.93)	0.49 (0.27–0.91)
High deprivation (8–10)	56/751 (7.5)	0.95 (0.56–1.59)		0.86 (0.48–1.51)	0.83 (0.48–1.45)
**ANY ETHNIC DISCRIMINATION**					
No	63/1,225 (5.1)	1			1
Yes	44/413 (10.7)	2.23 (1.59–3.14)			2.47 (1.71–3.58)
Total	107/1,638 (6.5)				

*Model 1, Unadjusted model for associations with WHO wellbeing; Model 2, Model adjusted for age and sex; Model 3, Model adjusted for age, sex and NZ Dep; Model 4, Model adjusted for age, sex, NZ Dep and discrimination*.

## Discussion

In this large, nationally representative study of indigenous Māori students from mainstream education, we found that a strong sense of a Māori cultural identity was associated with improved wellbeing and reduced serious depressive symptoms. Ethnic discrimination was found to confound the relationship between mental health wellbeing and cultural identity, and associated with poorer wellbeing, increased depressive symptoms and suicide attempts amongst Māori students.

These findings are supportive of earlier work that found a strong sense of cultural identity is protective for mental health outcomes (18, 19, 40). Indigenous peoples have always asserted that a strong sense of cultural identity and pride was intimately linked to wellbeing; but through colonization these have been, and continue to be devalued by dominant cultural perspectives (17, 28, 46). Ethnic discrimination was common (25.5%) amongst Māori students attending mainstream education in New Zealand, and associated with poorer mental health. The strong effect of discrimination and lesser influence of socio-economic deprivation for suicide attempts (medium level deprivation was protective) is an important finding and differs from previous literature (47). Previously research has emphasized socio-economic factors as a strong influence for health inequity in mental health outcomes for Maori; however our finding suggests that while socio-economic factors are important, experiences of discrimination may be more powerful in mediating mental health access. This has been previously reported among African American males, where discrimination was a more powerful indicator of suicide attempt than socio-economic status (48). We believe that ethnic discrimination is likely to be a strong contributor to the perpetual disadvantage in our health system among Māori populations (49–53). We recommend any future suicide prevention research must collect and analyse ethnic discrimination data to enhance explanatory understandings of suicide among indigenous and minority populations.

Of interest, is the finding that students who live with socio-economic deprivation have stronger WHO-5 wellbeing scores. It may be as Bécares et al. (41) found, that living in poorer communities that have high ethnic density was associated with positive wellbeing, and possibly attributed to reduced exposure to discrimination, increased social supports and increased access to various cultural resources. Conversely, poor students living in affluent neighborhoods experienced higher levels of depressive symptoms compared to poor students living in poor neighborhoods, reflecting the psychosocial stress of perceived socioeconomic incongruity as well as poorer access to social networks (54). Harris et al. (55) also reported that socially assigned ethnicity or being classified by others as “White” was associated with health advantage reinforcing that structural and interpersonal racism in healthcare is harmful.

The measurement of cultural and ethnic identity in quantitative research is challenging. As we reviewed the literature, we identified some concerning practices that were flawed theoretically and offensive to Māori researchers. For example, the practice of comparing “sole Māori” vs. “Māori/other identity” as a proxy for “strength of cultural identity” (21) has no theoretical or methodological foundation. Māori do not use “blood quantum” measures to define who we are or identify how strongly we identify as Māori. An example of this type of damaging ideology in well regarded research claims Māori specific cultural programming as “not evidence-based and largely ideologically driven” and concluded that “[e]ven though such policies are no doubt well intentioned and observe statutory requirements unique to the New Zealand context … they must be exposed to ongoing critical scrutiny and empirical evaluation” (21, p. 167). This example highlights that privileging of knowledge from dominant culture academia, using racist interpretations that systematically undermine Māori knowledge and experiences. These practices hinder efforts to introduce and expand effective kaupapa Māori strategies (incorporating the knowledge, skills, attitudes and values of Māori society) in the New Zealand context (56), and must be challenged through anti-racism praxis (57).

As with all cross-sectional studies, this study was unable to infer causality. A further limitation is that these findings cannot be generalized to all Māori youth, as those who attend Wharekura (Māori language immersion Secondary Schools), Alternative Education (58) or are not enrolled in any schooling are not represented in these data and are likely to have greater risks. Also of note, is that at age 16 years, students can leave school in New Zealand. Given Māori are more likely to drop out of education, it may be that our finding that younger students are at more risk may be an artifact due to older Maori students no longer being in full-time education. There are also limitations with the measurement of socio-economic deprivation utilizing the NZ Dep2006 as these are based on population level census and not sensitive to individual/family level measures. Finally, the measurement of Māori cultural identity is challenging to measure in quantitative research and in some cases has been successful (34). The Youth'12 survey had limited options available for the development of a comprehensive MCIS scale based on dimensions identified in the literature. In addition, while this is a nation-wide study, the cultural identities of youth in various rohe (areas/regions and tribal communities) throughout New Zealand may not be adequately represented in the MCIS measure. Further research is required to explore the nuanced and contemporary knowledge of Maori youth cultural identities and to broaden the identity scale to include the concepts of whanaungatanga (collectivist identities and relationships).

In conclusion, Māori youth mental health is complex and multi-dimensional with multiple contributing factors embedded in cultural, historical, spiritual, physiological, psychological, structural and social domains. Our findings suggest that public health programmes and services that genuinely seek to address equity for Māori youth, will ensure cultural programming and policies that are culturally and developmentally specific, as core components of any mental health and suicide prevention strategy. Given the high rates of suicide and persistent inequity, we must support the continued reclaiming of traditional Māori knowledge and practices by contemporary Māori youth to heal, connect and give meaning to their lives as highlighted in The Turamarama Declaration (59). In parallel, there must be structural commitment to disrupt the status quo research, policies, programmes and service provision that perpetuate discrimination and result in inequitable outcomes for Māori. *Mauri Ora*.

## Author contributions

AW conducted the literature review, led the identification of survey variables for MCIS scale, drafted the outline of the manuscript and contributed to the analysis and interpretation. TC was principal investigator for the Youth'12 study, had oversight of the literature review, data analysis, interpretation and led the writing of the paper, structure, editing and discussion. SL contributed to the overall design of the paper, led the analysis and interpretation of data and contributed to the discussion and overall paper. All authors contributed to drafting and TC and SL did critical revisions, approved the final version for publication, and agreed to be accountable for all aspects of the work in ensuring that questions related to the accuracy or integrity of any part of the work are appropriately investigated and resolved.

### Conflict of interest statement

The authors declare that the research was conducted in the absence of any commercial or financial relationships that could be construed as a potential conflict of interest.
